# Disparities in Access to Spinal Surgery Based on Insurance Type Among Elderly Patients

**DOI:** 10.7759/cureus.81053

**Published:** 2025-03-23

**Authors:** Jose G Lima, Timothy P Skerry, Alexander J Rodriguez, Mikahla E Gay, Juan C Alvarez, Peter W D'Amore

**Affiliations:** 1 Department of Medicine, Florida International University, Herbert Wertheim College of Medicine, Miami, USA; 2 Department of Orthopedic Surgery, Broward Health Medical Center, Fort Lauderdale, USA; 3 Department of Minimally Invasive and Robotic Spine Surgery, Orthopedic Center of South Florida, Miami, USA

**Keywords:** access to healthcare, elderly population, healthcare disparities, healthcare insurance, insurance type, lower back pain (lbp), orthopedic surgeon, social determinants of health (sdoh), spinal surgeon, spine injuries

## Abstract

Background: Spinal surgeries are increasingly performed in the aging United States Medicare population. There has been a demonstrated increase in the utilization of multiple core spine procedures over the last two decades in the over-65 age demographic. Costs for core procedures, such as spinal fusions, have doubled for government-based insurance programs. Additional barriers to access to care may lead to increased complication rates among patients with public insurance compared to private insurance carriers. With an increasing demand for spinal operations coinciding with the aging population, we seek to examine trends in access to spinal surgery for Medicare Advantage (MA) plans compared to private insurance and Traditional Medicare (TM) programs.

Methods: In this cross-sectional study, a search of the American Academy of Orthopedic Surgeons directory was conducted for spine surgeons practicing in Florida. Once identified, offices were contacted on four occasions by four separate researchers to assess insurance acceptance and appointment availability using the “secret shopper” technique. This technique utilized a script for a fictitious elderly patient seeking spinal fusion. Insurance plans assessed included TM, Blue Cross Blue Shield (BCBS), and two MA plans (Humana Choice Preferred Provider Organization, PPO, and United Health Preferred Choice Health Maintenance Organization, HMO). Appointment availability was measured in business days. Physicians who retired, relocated, no longer performed spinal fusion surgery, or could not be contacted were excluded from the study.

Results: Of the 120 contacted orthopedic spine surgeons, 55 surgeons met eligibility criteria and reported accepting new surgical consultations. Out of the 55 eligible surgeons, 27 (49%) confirmed in-network benefits for Humana Choice (MA-PPO), and 26 (47%) confirmed in-network benefits for United Healthcare Preferred Choice (MA-HMO). In comparison, BCBS BlueOptions and TM were in-network for 41 (74%) and 47 (85%) of eligible surgeons, respectively. Humana Choice and United Healthcare Preferred Choice were accepted at significantly lower rates than TM and BCBS BlueOptions (p < 0.01). Time to appointment did not differ based on insurance type (p = 0.575).

Conclusion: This study found that patients with MA plans have significantly decreased access to spine surgeons in the state of Florida compared to those with TM and private insurance. Our study highlights a trend in patients with MA plans that may put them at a disadvantage when it comes to patient care. Additional research is required to clarify the broader implications and financial impacts of variations in insurance provider accessibility.

## Introduction

Spinal surgeries are increasingly performed in the aging United States Medicare population. Data have demonstrated an 88% increase in the utilization of multiple core spine procedures over the last two decades in the over-65 age demographic [1]. One in five Americans is projected to reach retirement age by 2030, and the number of individuals 65 years and older is expected to nearly double by 2060, from 49 million to 95 million [1]. As the United States population ages, there is an expected rise in multilevel spinal surgery due to the increased prevalence of degenerative spinal disorders, including osteoporotic fractures, spinal stenosis, spondylolisthesis, and scoliosis [2]. Projected utilization of spinal fusions exceeds an 80% increase by 2060 [3].

In the past two decades, patients' insurance coverage has changed drastically. The Traditional Medicare (TM) fee-for-service model was used extensively, but in recent years, Medicare Advantage (MA) has seen an increase in beneficiaries. MA is commonly known as Medicare Part C, which provides patients with additional private medical insurance that pays for their care. The goal of MA plans is for patients to participate in the private insurance market to help supplement their costs that TM may not cover, such as dental or vision, as well as coordinate medical care by using a network within an insurance company [4]. MA plans can also be tailored to specific populations, such as disabled patients or those with chronic conditions. However, one of the main limitations of MA plans is this provider network constrains the patient from making choices about their physician, with the added hurdle of also needing referrals and authorizations before their care is covered. Anderson et al. discuss the substantial administrative and medical burden that MA prior authorizations cost to the healthcare system and patients, causing unnecessary delays and denials in access to medical care [5]. In 2022, 13% of 12,273 requests to MA plans were improperly denied despite meeting Medicare coverage requirements [6]. Despite these drawbacks, Jacobson and Blumenthal showed that almost half of the roughly 65 million Medicare beneficiaries participated in MA plans in 2022, with projected growth to increase that percentage of MA participants [7]. The potential reasons for this growth include higher rebates, limits on out-of-pocket spending, MA plan marketing, and simplicity in single plan coverage for all patients' needs [7].

With the aging population in the United States, there will be an increasing number of patients who have MA plans and are requiring spinal surgery. It is important to evaluate the impact of the differences in MA plans compared to TM on access to spine surgery. Spinal surgeries are typically performed by either orthopedic spine surgeons or neurosurgeons. As stated above, there can be many barriers to accessing these physicians in MA plans, including referral and prior authorization requirements. This study aims to evaluate if enrollment in MA plans affects access to orthopedic spine surgeons for spinal surgery compared to enrollment in TM or Private Insurance.

This article was previously presented as a meeting abstract at the Florida Medical Association’s David A. Paulus, MD Poster Symposium on August 3, 2024.

## Materials and methods

The American Academy of Orthopedic Surgeons directory was searched to find orthopedic spine surgeons practicing in Florida. Orthopedic spine surgeons who had retired, moved out of Florida, or no longer performed surgery were excluded. Each spine surgeon was assigned an identification number.

Three major types of insurance plans were studied: TM plans, MA plans, and Commercial insurance plans. Blue Cross Blue Shield (BCBS) of Florida was chosen as the private commercial insurance study, as it is the largest commercial insurer in Florida according to the Florida Agency for Health Care Administration. United Healthcare and Humana were selected as the MA representatives as they are among the top MA insurers in the United States [8]. The United Healthcare Preferred Choice (Health Maintenance Organization, HMO) MA plan and Humana Choice (Preferred Provider Organization, PPO) MA plan were chosen specifically.

Once the eligible orthopedic spine physicians and insurance plans were identified, the offices were contacted by phone to collect the data. Phone calls were made during business hours of 8 AM to 5 PM. Using a systematic script, the researcher would assess a single insurance acceptance and appointment availability using the “secret shopper” technique. The script simulated a real-life patient with the researcher calling on behalf of his/her father, a 65-year-old male patient with chronic low back pain, requesting a new consultation for potential lumbar fusion surgery. The researchers were instructed not to book actual appointments but to collect the necessary information. The data, including acceptance of insurance, number of business days until the earliest appointment, and any reasons the patient could not be seen, was collected on a Microsoft Excel Sheet (Microsoft Corporation, Redmond, WA). Acceptance of insurance was determined by individual office responses, and surgeons were categorized as either accepting or not accepting each insurance plan. The offices were contacted on four occasions by four individual researchers to inquire about each specific insurance plan and prevent voice recognition.

A protocol was created for researchers when it was difficult to contact an office. A researcher would wait on hold for no longer than 10 minutes before ending the call. Offices requiring an extended hold time or voicemail were called again on up to four separate occasions at later dates to attempt to collect the data. Physicians whose offices could not be contacted on five separate occasions were excluded from the study. Sixty-five physicians met these criteria and were excluded from the study.

This study did not use any human subjects or patient data. Due to this, informed consent was not required, and International Review Board Approval was not needed.

## Results

**Figure 1:**
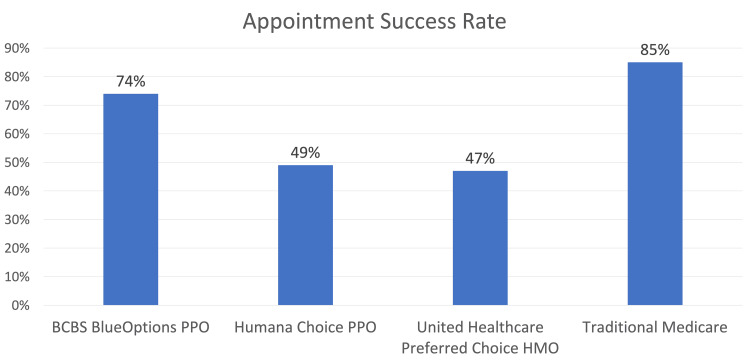
Chart showing the physician acceptance rate for each insurance type BCBS: Blue Cross Blue Shield; PPO: Preferred Provider Organization; HMO: Health Maintenance Organization

Of the 120 contacted Florida orthopedic spine surgeons, 55 surgeons met eligibility criteria and reported accepting new surgical consultations. Out of the 55 eligible surgeons, 27 (49%) confirmed in-network benefits for Humana Choice (MA-PPO), and 26 (47%) confirmed in-network benefits for United Healthcare Preferred Choice (MA-HMO). In comparison, BCBS BlueOptions and TM were in-network for 41 (74%) and 47 (85%) of eligible surgeons, respectively (Figure 1). Humana Choice and United Healthcare Preferred Choice were accepted at statistically significantly lower rates than TM and BCBS BlueOptions (p < 0.01). When Humana Choice and United Healthcare Preferred Choice were compared, there was no statistically significant difference in acceptance (p = 0.849). Comparing BCBS BlueOptions and TM also showed no statistically significant difference in acceptance (p = 0.157).

**Figure 2:**
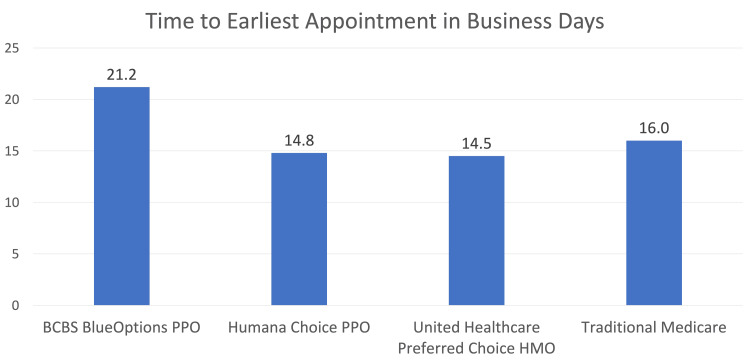
Chart showing average time (business days) to next available appointment BCBS: Blue Cross Blue Shield; PPO: Preferred Provider Organization; HMO: Health Maintenance Organization

When analyzing the average time to appointment, BCBS had 21.2 days (SD = 27.4), Humana had 14.8 days (SD = 16.3), United Healthcare had 16.0 (SD = 25.7), and TM had 14.5 (SD = 14.8) (Figure 2). The average time to appointment did not differ statistically based on insurance type (p = 0.575).

## Discussion

In this study, we employed the secret shopper methodology to acquire data about access to orthopedic spine surgeons in Florida, focusing on potential disparities among elderly patients with lower back pain covered by different insurance plans. Specifically, we investigated the impact of TM, private insurance, and MA coverage on access to care. Florida, with the second-largest elderly population in the United States, was an ideal setting for our research due to its demographic composition and diverse medical insurance landscape [9]. As of 2022, Florida ranks among the top five states for MA enrollment, which has risen dramatically from 6.3 million beneficiaries in 2006 to 30.3 million in 2022 [10,11]. The aging population and subsequent increases in MA enrollment underscore the importance of understanding how insurance type influences access to care.

Our findings revealed a significant disparity in access, with patients with TM and private insurance plans obtaining an appointment with orthopedic spine surgeons at higher rates than MA plans. This discrepancy in access to care aligns with previous studies, such as the work by Cifuentes et al., which also identified barriers to care for MA beneficiaries in accessing pain management specialists [12]. These findings emphasize a concern in the elderly population, as inadequate access to care can lead to unnecessary progression of life-threatening conditions such as cauda equina syndrome, vertebral osteomyelitis, or malignancy. It also raises concern because of the increasing enrollment in MA plans nationwide, which may be contributing to delayed care in the elderly population. Studies have hinted at the decreased access to care with MA plans compared to other insurance plans, and our study adds to the concern [12,13].

The differential diagnosis of lower back pain in an elderly patient is extensive, encompassing conditions such as degenerative disc disease, spinal stenosis, and muscle strain. Prompt evaluation and management are necessary when dealing with symptoms of lower back pain. Studies have shown that lower back pain is one of the most common conditions encountered worldwide, and incidence increases with age [14]. Furthermore, studies have shown that chronic lower back pain may become a disabling medical condition, limiting patients' ability to walk and function independently [15]. Adequate and timely care is essential for this population, and our study shows that the type of insurance coverage may be a barrier to care. Our study highlights an area of concern within public health and health literacy. It highlights the importance of understanding what type of insurance to obtain and the risks and benefits of each plan. It also shows the importance of insurance companies' transparency in equipping patients with the knowledge to choose the correct plan based on their medical concerns and needs. Equitable and timely care is something that all insurance beneficiaries should expect with their insurance plans.

Despite the significant difference in access to health care between TM, private insurance, and MA plans, certain limitations warrant consideration. One of the important limitations is that our study design did not allow for an assessment of why MA plans were accepted at lower rates than TM or private insurance. One potential reason for this observation is lower reimbursements with MA. Studies have proposed that MA plans may have a lower reimbursement rate than other insurance plans for the same procedures [16]. Another potential reason for discrepancies is that most MA plans require referrals to obtain an appointment with a specialist, such as an orthopedic spine surgeon. This delays and inherently reduces access to appointments.

Additionally, our study design may be considered a limitation. Since our study was a cross-sectional study, we analyzed access to appointments at one point in time, but we could not study appointment costs, co-pay, patient experience, or appointment availability over time. This limited the focus of our study to healthcare barriers upon entrance to a new practice. Additionally, in our study, we reviewed four different types of insurance, which may not be representative of all insurance plans within these categories; however, efforts to include various insurance companies and plan types mitigate the potential bias of this limitation. Finally, we specifically assessed access in Florida, and 55 of the original 120 physicians (45%) met the inclusion criteria, decreasing our sample size and generalizability across the United States. While these limitations exist, our data were representative of physicians across the state of Florida, which encompasses various racial, ethnic, and minority populations. Thus, the generalizability should not be limited by the geographic or inclusion criteria.

Future studies should expand geographically and incorporate patient experience, cost of visit, and reimbursement of procedure based on the insurance companies. This will help create a more complete picture of the factors affecting access to orthopedic spine surgeons in elderly populations nationwide.

## Conclusions

This cross-sectional analysis demonstrated decreased access to spine surgeons for Humana and United Healthcare MA plans compared to TM and BCBS insurance plans. This study demonstrates a concerning trend with MA plans and access to healthcare, impeding access to care and orthopedic spine treatment for an aging patient population. Further research is needed to elucidate clinical implications, associated costs, and potential solutions for differences in insurance provider and accessibility nationwide.
